# Influence of Self-Reported Chronic Rhinosinusitis on Health-Related Quality of Life: A Population-Based Survey

**DOI:** 10.1371/journal.pone.0126881

**Published:** 2015-05-15

**Authors:** Qing-Ling Fu, Jin-Xiang Ma, Chun-Quan Ou, Cui Guo, Shuang-Quan Shen, Geng Xu, Jianbo Shi

**Affiliations:** 1 Otorhinolaryngology Hospital, The First Affiliated Hospital, Sun Yat-sen University, 58 Zhongshan Road II, Guangzhou, Guangdong, 510080, China; 2 Department of Applied Statistics, School of Public Health, Guangzhou Medical University, Guangzhou, Guangdong, 511436, China; 3 State Key Laboratory of Organ Failure Research, Department of Biostatistics, School of Public Health and Tropical Medicine, Southern Medical University, Guangzhou, Guangdong, 510515, China; Tongji Medical College, CHINA

## Abstract

Chronic rhinosinusitis (CRS) is a frequently occurring chronic respiratory disease. There is evidence that effective treatment of CRS can improve patients’ quality of life, but the data regarding the extent to which CRS impairs patients’ quality of life (QoL) is sparse. This study aimed to evaluate the effect of self-reported CRS on health-related QoL and to determine whether the influence was associated with gender, age and socio-economic status. A four-stage random sampling method was used to select the participants from the general population in Guangzhou, China. All participants were interviewed face-to-face at their homes using a standardized questionnaire. The health-related QoL of each participant was assessed using the SF-36 Health Survey. The scores of the SF-36 after adjusting for gender, age, socioeconomic conditions, smoking and some important comorbid conditions were compared between the CRS group and the non-CRS group using analysis of covariance. A multiple linear regression model with interaction terms was established to determine whether CRS affected QoL to the same degree across the different subpopulations. Among a total of 1,411 participants aged at least 15 years, 118 persons (8.4%) had self-reported CRS. Subjects with CRS had an increased prevalence of allergic rhinitis, chronic obstructive pulmonary disease and gout than subjects without CRS. The CRS group had lower scores in all eight domains and the physical and mental component summary than those without CRS (P<0.05), and the greatest differences were in role emotional function (RE), general health (GH) and role physical function (RP). The impairments of the CRS participants in RE and RP were greater among the females than the males. Moreover, physical domains were affected to greater degrees among the elderly and those with high-level education. In conclusion, CRS is a common chronic disorder. Persons with self-reported CRS perceived themselves as having impaired QoL in both the physical and mental domains. These findings shed new light on the health burden of CRS and should be taken into account by clinicians involved in the care of CRS patients.

## Introduction

Chronic rhinosinusitis (CRS) is one of the most common chronic conditions with estimated prevalences of 10.9% in Europe [1] and 16% in the USA [2]. Despite the extensive studies on CRS, many uncertainties remain regarding its cause, pathophysiology and optimal treatment. Some studies have demonstrated the enormous economic burden of CRS in terms of direct health costs and losses of productivity [3,4]. CRS brings about significant physical symptoms, such as nasal blockage, rhinorrhea, a reduced sense of smell, facial pain or pressure and headache, which are persistent although not fatal and result in considerable negative influences on patients’ daily lives and emotions. CRS-associated loss of quality of life (QoL) is becoming increasingly concerning. Specifically, from the patient’s perspective, the manner in which CRS affects daily life is far more important than the results of medical examinations such as CT scans [5]. Health-related QoL, which integrates the physical, social and psychological effects of an illness on a patient, has been widely used as an important variable for measuring the severity of symptoms and evaluating the effectiveness of therapies for CRS [6].

A number of clinical studies have shown that adequate treatment of CRS can contribute to significant improvements in QoL [5,7–10], which suggests that CRS impairs QoL. However, there are few population-based studies of the effects of CRS on QoL partly due to a lack of a generally accepted symptoms-based CRS definition. Previous studies of the QoLs of CRS patients have focused on CRS patients who were recruited from the otorhinolaryngology departments and diagnosed by clinical examination (e.g., with nasal endoscopies and CT scans) and thus focused on those with serious symptoms. A recent study by Lange et al. [11] evaluated the QoL of CRS patients by comparing persons with and without CRS who attended clinical visits. Some people with relatively mild symptoms of CRS who might not visit physicians remain, and it is unknown whether and to what extent such people experience impaired quality of life. Additionally, the controls selected from clinical visits or inpatients without CRS but with some other disorders tend to have lower qualities of life than the general population; therefore, this selection bias likely results in underestimations of the effects of CRS on quality of life. Furthermore, whether CRS impairs QoL to the same degree in different subpopulations has not been determined.

The symptom-based definition of CRS provided in European Position Paper on Rhinosinusitis and Nasal Polyps (EP^3^OS) has been validated and is suitable for use in epidemiological and clinical research [1,12–14]. This definition allowed us to thoroughly assess the QoL of CRS patients in a general population. We conducted a cross-sectional household survey in Guangzhou, China. This population-based study aimed to quantify the effect of self-reported CRS on QoL and further determine the potential differences in this effect due to gender, age and socio-economic status.

## Materials and Methods

### Data collection

The participants were selected from the general population in Guangzhou using a four-stage random sampling method. The sampling method was described in details in Chinese multiple-city CRS investigation in which Guangzhou was involved as one of the seven cities under study[15]. In brief, we obtained the list of districts, streets and communities of Guangzhou. Firstly, we randomly selected two districts from six central urban districts in Guangzhou. Next, we used simple random sampling to select two streets within each selected district and two communities from each selected street. In the final stage, we randomly chose 53 households in each of 8 selected communities. The subjects included all Chinese residents of chosen households who were aged 15 years or above and had lived in Guangzhou for at least one year at the time the study was conducted (S1 File).

All subjects were interviewed face-to-face at their own house, and they were asked to complete a self-administered questionnaire (S2 File). The questionnaire covered socioeconomic and demographic characteristics, and the interviews included questions about CRS-related symptoms and information about health-related QoL as assessed by the Chinese standard version of the SF-36. The reliability and validity of the Chinese version of the SF-36 was confirmed in many studies and has been widely used to measure QoL of patients and healthy general population in China [16–18]. The SF-36 consists of 36 items and measures health status in eight domains, including physical functioning (PF), role physical function (RP), bodily pain (BP), general health (GH), vitality (VT), role emotional functioning (RE), social functioning (SF), and mental health (MH).

### Data analysis

Double data entry and consistency checking were performed with Epidata 3.1. All data were analyzed using SPSS for Windows 19.0. We compared the characteristics between the groups with and without self-reported CRS using chi-square tests. According to the manual for the SF-36 Health Survey [19], the items of the SF-36 were transformed and summed to a 0–100 scale for each domain in which higher scores indicated a better health-related QoL. We used analyses of covariance to compare domain-specific SF-36 scores between CRS and non-CRS groups after adjusting for gender, age, education level, smoking, monthly household per capita income and self-reported medical history of some important comorbid conditions, including asthma, allergic rhinitis, chronic obstructive pulmonary disease (COPD) and gout. Differences with P values below 0.05 according to two-tailed tests were regarded as statistically significant. A multiple linear regression model of the SF-36 scores was established with the interaction between the above-mentioned socio-demographic factors and CRS as the indicator variable to determine whether CRS affected QoL to the same degree across the different subpopulations.

### Ethic statement

The study protocol was approved by the Ethics Committee of the First Affiliated Hospital, Sun Yat-sen University (No. 201432). The interviewer explained the purpose of the investigation and the procedures and acquired written informed consent from each subject or their guardians on behalf of the children enrolled in the study before commencing the interview.

## Results

Among the random sample of 1,534 subjects of at least 15 years of age, 1,411 responded and completed the questionnaire for a valid response rate of 92%. The mean age of the participants was 45.8 years (SD = 19.7), and females accounted for 51.2% of all of the participants.

The participants provided information about CRS symptoms. A symptoms-based diagnosis of CRS was made if the person presented with at least two of the following four symptoms for more than 12 weeks in the previous one year and at least one of the first two symptoms were present: nasal obstruction/blockage/congestion, nasal discharge (anterior/posterior/nasal drip or purulent throat mucus), facial (forehead/nasal/eye) pain/pressure, and reduction or loss of smell. Consequently, 118 persons had self-reported CRS for an overall prevalence of 8.4% (95% confidence interval: 7.0%-9.9%). This prevalence did not significantly differ between the males and females (9.0% vs. 7.7%, P = 0.390).

**Fig 1:**
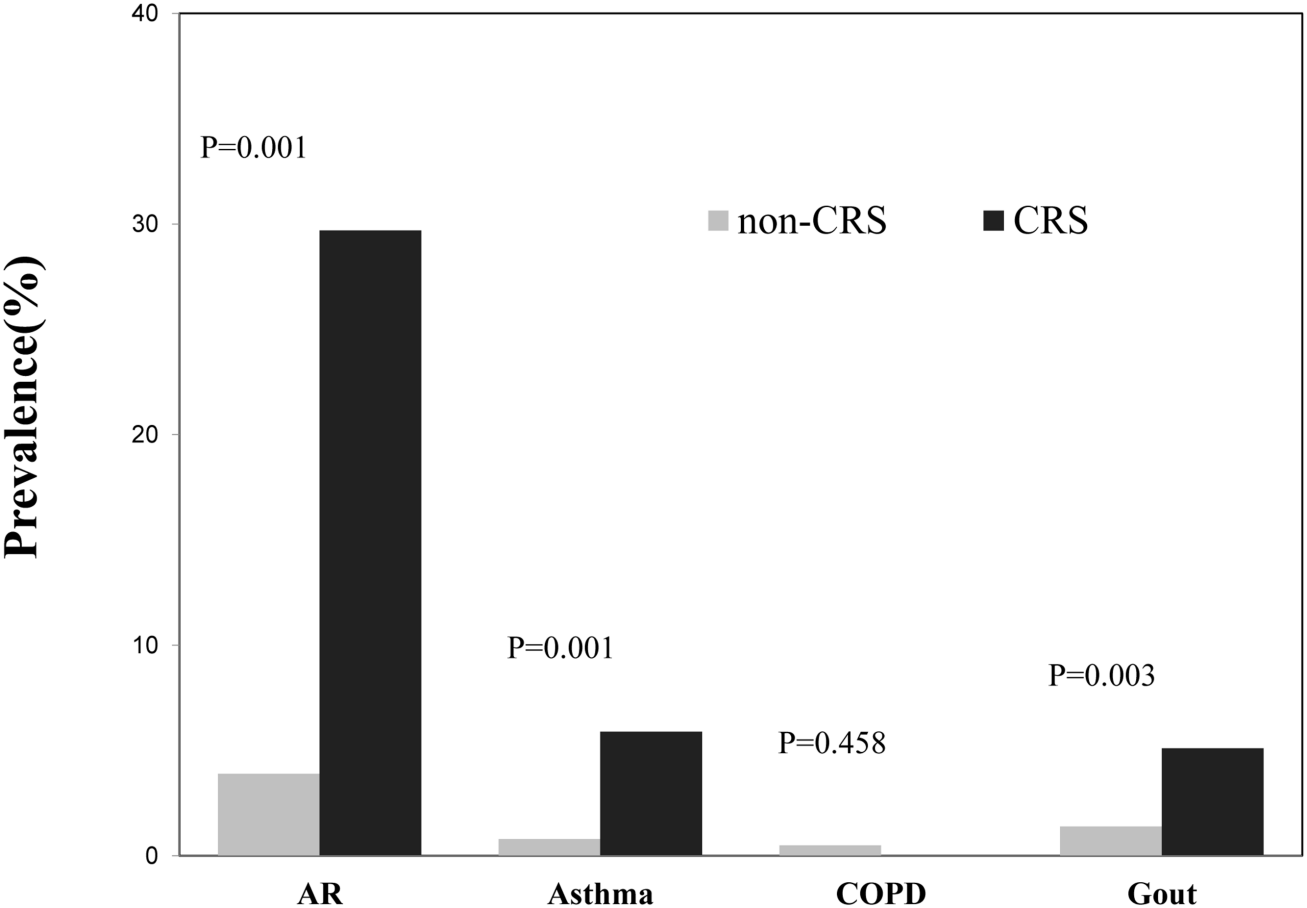
The prevalence of a comorbid condition in the chronic rhinosinusitis (CRS) group and the non-CRS group. AR: allergic rhinitis; COPD: chronic obstructive pulmonary disease (COPD).

There were no significant differences in age, gender or monthly household per capita income between the CRS group and the non-CRS group, but the subjects with CRS tended to have higher levels of education level (Table 1). The prevalence of a self-reported medical history of asthma, allergic rhinitis and gout was significantly higher in the CRS group than that in the non-CRS group (Fig 1).

**Table 1:** Comparison of characteristics of the chronic rhinosinusitis (CRS) group and non-CRS group.

Characteristic		Non-CRS	CRS	P value
		n(%)	n(%)	
**Gender**	Male	626(48.4)	62(52.5)	0.390
	Female	667(51.6)	56(47.5)	
**Age(years)**	<30	380(29.4)	41(34.7)	0.269
	30–59	591(45.7)	45(38.1)	
	60 or above	322(24.9)	32(27.1)	
**Monthly mean**	<RMB $1000	166(12.9)	11(9.3)	0.460
**household income**	RMB $1001–3000	880(68.1)	81(68.6)	
	RMB $3000+	247(19.2)	26(22.1)	
**Education**	Illiterate and primary school	202(15.7)	19(16.1)	0.001
	Secondary school	343(26.5)	22(18.6)	
	High school and above	748(57.8)	77(65.3)	

**Fig 2:**
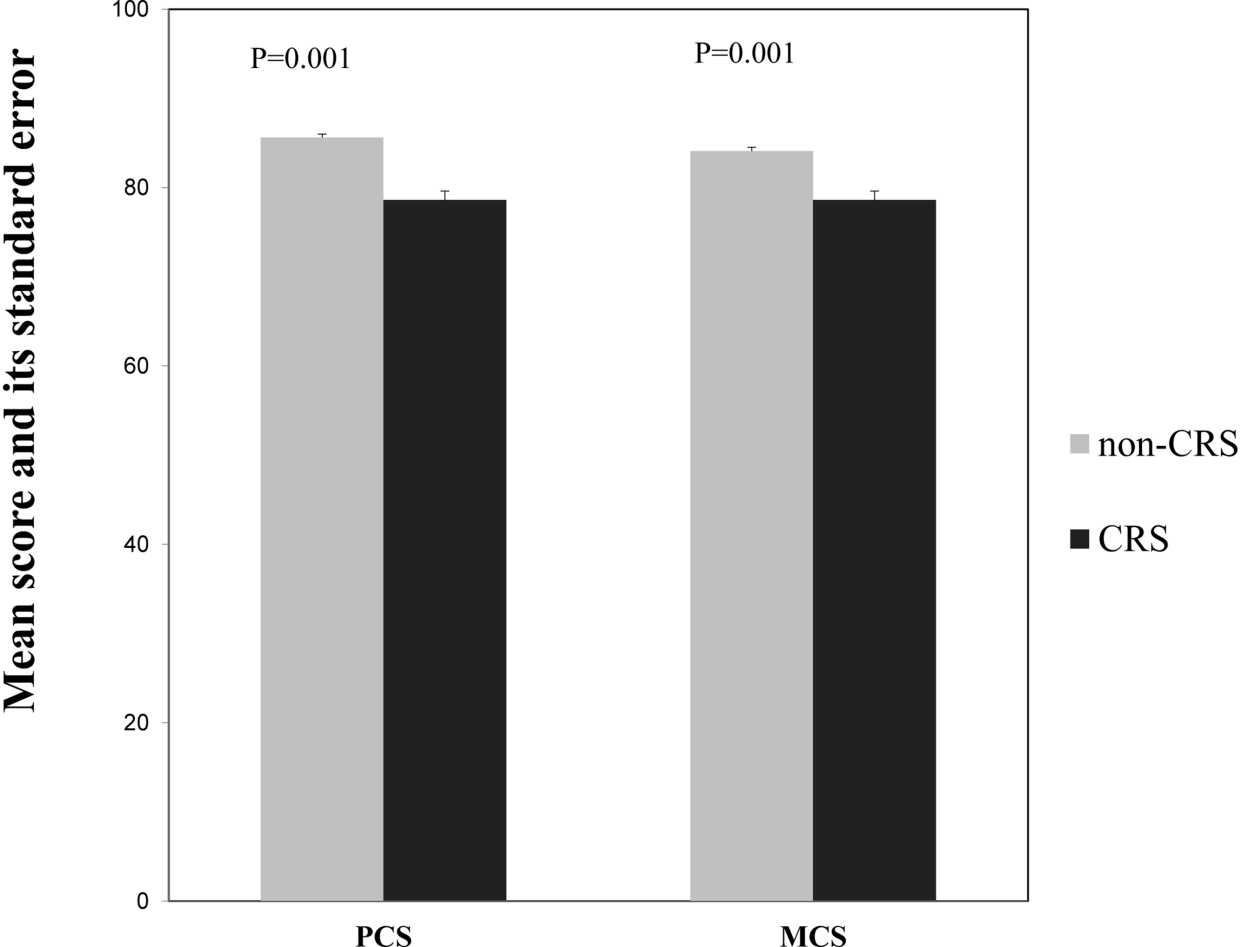
The comparison of the physical and mental component summary score of the SF-36 between the chronic rhinosinusitis (CRS) group and the non-CRS group.

After adjusting for gender, age, education level, household per capita income, smoking and the above-mentioned comorbid conditions, the average scores for each of the eight SF-36 domains were significantly lower in the CRS group than in the non-CRS group (P<0.05), and the greatest differences of approximately 10 points were observed in role emotional function, general health and role physical function. The differences in physical functioning, mental health and vitality were relatively small; an average decrease across these scores of 2–4 in the CRS group compared to the non-CRS group was observed (Table 2). Compared to the non-CRS group, the CRS group had a significant decrease of 7.0 and 5.5 in the mean score of the physical (PCS) and mental component summary (MCS), respectively (Fig 2).

**Table 2:** Comparison of the SF-36 scores between the chronic rhinosinusitis (CRS) group and non-CRS group.

Domain	Non-CRS	CRS	Difference	P value
	(mean±SEM)^a^	(mean±SEM) ^a^	(mean±SEM) ^a^	
PF	93.6±0.3	90.8±0.9	2.8±1.0	0.004
RP	91.8±0.6	83.9±2.2	7.9±2.4	0.001
BP	92.8±0.3	87.7±1.2	5.1±1.2	0.001
GH	64.1±0.4	55.1±1.4	9.0±1.5	0.001
VT	75.6±0.4	71.2±1.2	4.4±1.3	0.001
SF	89.0±0.4	82.6±1.3	6.4±1.4	0.001
RE	93.4±0.6	83.6±2.1	9.8±2.2	0.001
MH	78.7±0.3	75.3±1.1	3.4±1.2	0.004

^**a**^ The means adjusted for gender, age, smoking, education and mean monthly household income were calculated and compared using analyses of covariance.

Abbreviations: PF, physical functioning; RP, role physical function; BP, bodily pain; GH, general health; VT, vitality; SF, social functioning; RE, role emotional function; MH, mental health.

There were no significant interactions between gender and CRS in any of the domains of the SF-36 with the exceptions of role physical function and role emotional function; the impairments in the mean scores for these domains due to CRS were 11.6 and 13.7 greater, respectively, among females than males. The effect of CRS on three physical domains (PF, RP and BP) consistently varied with age and education; greater effects were present in the elderly and those with high levels of education. The CRS-associated impairment in general health and role emotional function was also observed to increase with education level, while the effects of CRS did not seem to differ with household per capita income in any of the domains. For the SF-36 summary component, both PCS and MCS score were significantly lower in females and those with high education level. In addition, the PCS score but not the MCS score significantly decreased with age (Table 3).

**Table 3:** Interactions between chronic rhinosinusitis (CRS) and socio-demographic characteristics on the SF-36 score (n = 1,411).

Domain	Regression coefficient of interaction, b (P value)^a^			
	Sex	Age	Education	Income
**PF**	-2.9(0.182)	**-6.9(0.001)**	**-4.5(0.004)**	-2.4(0.208)
**RP**	**-11.6(0.016)**	**-12.3(0.001)**	**-11.2(0.001)**	4.7(0.265)
**BP**	-4.2(0.092)	**-3.9(0.013)**	**-4.6(0.011)**	1.2(0.578)
**GH**	-2.9(0.352)	-3.0(0.118)	**-4.6(0.011)**	1.2(0.578)
**VT**	-3.8(0.140)	-1.8(0.261)	-2.9(0.163)	-3.4(0.140)
**SF**	0.8(0.775)	-1.3(0.459)	-0.8(0.692)	3.6(0.154)
**RE**	**-13.7(0.002)**	-2.7(0.328)	**-13.3(0.001)**	-1.0(0.789)
**MH**	-1.3(0.593)	1.4(0.348)	-2.2(0.207)	-2.4(0.254)
**PCS**	**-5.4(0.021)**	**-6.5(0.001)**	**-7.0(0.001)**	0.2(0.940)
**MCS**	**-4.5(0.043)**	-1.1(0.427)	**-4.7(0.003)**	-0.8(0.681)

^**a**^ The regression coefficients of the interactions between CRS and each specific factor on each domain are presented and were statistically tested.

Abbreviations: PF, physical functioning; RP, role physical function; BP, bodily pain; GH, general health; VT, vitality; RE, role emotional function; SF, social functioning; MH, mental health; PCS, physical component summary; MCS, mental component summary.

In addition to the evaluation of general health status with the SF-36, we investigated other aspects of the health burden of CRS. Less than half (44.9%) of the subjects with self-reported CRS had visited physicians due to rhinitis or rhinosinusitis within the previous year. On average, the subjects had visited physicians 4.5 times and had missed 11.7 days of work or school in the previous one year. All subjects reported their quality of sleep on a 1–5 scale. The persons with CRS exhibited worse self-reported quality of sleep than did those without CRS (Z = 2.647, P = 0.008). Moreover, the CRS group was more likely to wake up at night due to shortness of breath or coughing than the non-CRS group (6.9% vs. 1.4% and 14.7% vs. 4.2%, respectively, P<0.001).

## Discussion

The questionnaire survey of a random sample of 1,411 participants from the general population revealed an 8.4% prevalence of self-reported CRS in Guangzhou. This level is slightly lower than the estimate of 10.9% in Europe [1]. We observed repeated medical visits and losses of work days associated with CRS, indicating a remarkable economic burden of CRS.

There is an increasing interest in assessing patients’ QoL. Many different generic and specific questionnaires have been validated and used to evaluate QoL in patients with CRS [6,20]. In this population-based survey, we used the SF-36 to measure health-related QoL. The SF-36 was developed from the Medical Outcome Study in 1988 [19]. The SF-36 has been recognized as a valid and reliable instrument for the assessment of health status. It has been translated into many different languages, including Chinese, and is by far the most widely used generic scale of QoL. The most significant advantage over specific questionnaires, such as the Sino-Nasal Outcome Test 20 (SNOT-20), is that it is applicable to all health conditions, which allowed us to compare QoL between CRS patients, healthy subjects and patients with other diseases.

Nilsson et al. [21] demonstrated gender difference in SF-36 scores. These authors showed that the SF-36 scores of both healthy and unhealthy population vary by age and socio-economic status [22,23]. Our data for the CRS subjects also confirmed that health-related QoL varies by gender, age and education. Furthermore, an educational difference between the CRS and non-CRS group was identified, and there were also some age and gender differences that did not reach statistical significance. There are some comorbid medical conditions with CRS. Our data confirms an increased prevalence of asthma, AR and gout in subjects with CRS as compared to those without CRS. Therefore, to control for the potential confounding, we compared the QoL scores between the CRS group and the non-CRS group after adjusting for socio-demographic factors and these comorbid conditions [24]. The results indicated that CRS indeed impairs patients’ QoL CRS and this impairment is not due to its comorbid conditions. Gliklich and Metson [7] demonstrated that CRS patients who were waiting for nasal surgery exhibited significant lower SF-36 scores in five of the eight domains than did the American general population. A few other studies have also reported QoL impairments in all SF-36 domains with the exception of physical functioning in clinical-diagnosed CRS inpatients or outpatients relative to the normal population [8,25]. To our knowledge, this is the first study to examine the effects of self-reported CRS on quality of life in a general population, particularly in Asia; this study thus strengthens the evidence base regarding assessments of the health burden of CRS. As expected, the QoL scores of the self-reported CRS patients in our study were generally better than those of inpatients and outpatients with clinically diagnosed CRS as reported in a Chinese clinical trial [26], while the subjects with self-reported CRS exhibited significantly lower QoL scores across all physical and emotional domains compared to those of the subjects without CRS. These findings confirm that CRS affects people in multiple ways.

Guilemany et al. [27] demonstrated male patients with bronchiectasis exhibit better QoL scores in the domains of physical and social functioning than do females with bronchiectasis. A similar gender difference has been observed in patients with asthma [28]. However, no previous study has examined gender and age differences in the effects of CRS on QoL. In the present study, we found clear evidence that CRS affects QoL to different degrees in different subpopulations. CRS impaired role physical function and role emotional function to greater degrees in females than in males. The elderly and those with high levels of education reported greater impairments in all four physical domains. These findings will help to tailor subpopulation-specific education and management plans for CRS.

Despite the considerable effect of CRS on QoL, these impairments are fortunately reversible. A few studies have demonstrated that significant improvements in the QoL scores of CRS patients can be achieved following either medical or surgical treatments and that patients’ QoL scores can reach the normal levels of the general population 3–12 months following endonasal sinus surgery [7–9]. These findings suggest that early optimal treatment is beneficial to the recovery of QoL impairments.

Some studies have demonstrated the effects of allergic rhinitis on sleep quality and found that such impairments are strongly correlated with disease severity [29–31]. A few studies have also demonstrated sleep improvements following endoscopic sinus surgery in patients with CRS [32,33], but there has been few population-based studies that have assessed sleep quality in CRS patients. In the present study, we found that CRS remarkably impaired sleep quality, which might have subsequent negative effects on daytime performance and health-related QoL. To improve breathing and thus sleep, it is recommended that CRS patients, particularly those with serious symptoms of nasal blockage, use nasal steroid sprays before sleeping.

There are some limitations to this study. The literature suggests that self-reported QoL assessments are not merely measures of absolute function but also depend on people’s perceptions of their abilities [21]. This survey was designed to investigate CRS and health-related QoL, and those with CRS might tend to report inaccurately poorer QoL scores. CRS with and without nasal polys (e.g. CRSwNP and CRSsNP) are two important subtypes of CRS. According to EP^3^OS, nasal polyps should be defined by clinical criteria supported with endoscopy. In the present epidemiologic study, we collected medical history information but not clinical data of CRS subjects. Only 6 out of 118 subjects with CRS reported doctor-diagnosed nasal polyps. This self-reported history of doctor-diagnosed nasal polyps would provide a significant underestimate of the proportion of CRSwNP, because many patients do not see a doctor for nasal complaints in mainland China [15]. Because of the limited and underestimated number of CRSwNP, we did not make the comparison of QoL between CRSwNP and CRSsNP. Moreover, it remains unknown whether the impairments increased with the severity of CRS. Further studies using CRS-specific questionnaire, particularly clinical trials on CRS patients are required to analyze the potential correlation of QoL and disease severity and to compare the QoL between different subtypes of CRS.

In conclusion, this is the first study to investigate the relationship of QoL with self-reported CRS. CRS is a common respiratory disease that affects 8.4% of general population in Guangzhou, China. The persons with self-reported CRS perceived themselves as having impaired QoL in both physical and mental domains. These impairments were particularly severe in females, the elderly and those with high levels of education. These findings should be taken into account when evaluating the burden of CRS and considered by clinicians involved in the care of CRS patients.

## Supporting Information

S1 File Full raw dataset of the survey. (XLS)

S2 File The original Chinese form of questionnaire except SF-36 used in this study. (DOC)
