# Supplementary material for: Influence of Self-Reported Chronic Rhinosinusitis on Health-Related Quality of Life: A Population-Based Survey
Source: PLoS One. 2015 May 15;10(5):e0126881. doi: 10.1371/journal.pone.0126881 (PMC4433264; doi:10.1371/journal.pone.0126881)
Supplement: S2 File — (DOC) [file pone.0126881.s002.doc]

卫生部行业专项研究课题

编号：

慢性鼻窦炎问卷调查

慢性鼻窦炎是耳鼻喉科中的常见病、多发病，严重影响患者的日常生活和健康。为进一步摸清我国居民慢性鼻窦炎的发生发展规律以及与生活方式、职业暴露、环境等各种因素的关系，卫生部设立重点科技攻关项目，同时在广州、北京、上海等10个城市开展本次调查研究，本研究将为慢性鼻窦炎的有效防治提供科学依据。下面我们将占用您10～15分钟的时间，通过问卷的方式了解您个人的一般状况、鼻窦炎等病史、饮食、饮酒、吸烟习惯等情况。我们将对您提供的信息严格保密，所有研究结果只作为科学研究之用。对您的积极配合我们表示衷心的感谢！

**填写注意**:请在对应的数字序号上打“√”，在横线上填具体内容。__________________________________________________________________

**第一部分 基本情况**

**以下内容由调查工作人员填写**：

地址： 市 区 街道

居委会

被调查家庭编号：□□□□□

调查员姓名：­­­­­­­­­­­­­­­­­­­­­­

时间： 年 月 日

1. 性别： ①男 ②女
2. 民族： ①汉族 ②其他
3. 您目前的年龄（实岁）：　 岁; 身高 厘米; 体重 斤
4. 婚姻状况：①已婚 ②离婚 ③丧偶 ④未婚
5. 居住地性质：①城市 ②乡镇 ③农村
6. 居住情况：①与家人同住 ②与朋友同住 ③独居 ④其他
7. 您目前的住处住了 人，卧室有 间,住房总面积 平方米
8. 您目前的工作状况（勾选1个答案）：

| ①自己做生意 | ②上班族 | ③下岗 | ④因健康原因无法工作 |
| --- | --- | --- | --- |
| ⑤家庭主妇 | ⑥学生 | ⑦退休 | ⑧其它 |

1. 您目前如果在工作，工作性质是什么？（勾选1个答案）

①专业人员 ②管理人员 ③技术工人 ④半技术工人 ⑤非技术工人 ⑥其他

1. 您是否从事卫生服务工作（如医护人员、急救人员等）：① 否 ② 是
2. 您是否从事与清洁卫生有关的工作？ ①否 ② 是
3. 您的教育程度：

①文盲/没上过小学 ②小学 ③初中 ④高中/中专 ⑤大专或本科 ⑥研究生

1. 您父亲的教育程度：

①文盲/没上过小学 ②小学 ③初中 ④高中/中专 ⑤大专或本科 ⑥研究生

1. 您母亲的教育程度：

①文盲/没上过小学 ②小学 ③初中 ④高中/中专 ⑤大专或本科 ⑥研究生

1. 医疗费用支付类型：

| ①公费医疗保险 | ②农村合作医疗保险 | ③商业保险 |
| --- | --- | --- |
| ④社会医疗保险 | ⑤自费 | ⑥其它 |

1. 家庭人均月收入：

| ①300元以下 | ②301－1000元 | ③1001－3000元 |
| --- | --- | --- |
| ④3001－10000元 | ⑤10001－20000元 | ⑥20000元以上 |

**第二部分 鼻窦炎及相关疾患调查**

1． 在过去的1年中，您是否有**超过3个月**的时间出现以下症状呢？

1.1鼻塞 ①否 ②是

1.2前额部、鼻部或眼部的疼痛或压迫感 ①否 ②是

1.3脓性的鼻腔分泌物（鼻涕）或脓性的喉咙粘液 ①否 ②是

1.4感到嗅觉下降或丧失 ①否 ②是

1.5您是否因上述问题用过鼻腔喷雾剂呢？ ①否 ②是

1.6您是否因上述问题吃过消炎药呢？ ①否 ②是

2． 有关鼻的疾病中，曾有医生告诉您患有以下疾病吗？（**可多选**）

①慢性鼻窦炎 ②慢性鼻炎 ③过敏性鼻炎 ④鼻息肉

⑤其他 ⑥都没有 ⑦不清楚

有关鼻的疾病中，您的家人曾经患有以下疾病吗？（**可多选**）

①慢性鼻窦炎 ②慢性鼻炎 ③过敏性鼻炎 ④鼻息肉 ⑤其他 ⑥都没有 ⑦不清楚

3． 过去一年中，您因为鼻炎或鼻窦炎的问题看过医生吗？ ①否 ②是，总共看过 次医生。

4. 过去一年中，您曾经因为鼻炎或鼻窦炎的问题误工或者误课吗？ ①否 ②是，总共 天。

5. 您以前做过鼻部手术吗？①否 ②是，手术时间为： 年 月，手术原因是 。

6． 您经常(或在特定的季节内每天有1小时以上的时间)出现下列鼻子过敏症状吗？（**可多选**）

①清水样鼻涕 ②打喷嚏、尤其是强烈和阵发性的 ③鼻塞 ④鼻痒

⑤结膜炎（眼睛痒、红） ⑥其他 ⑦都没有 **请转问题7.**

6.1您有过1周内超过4天的时间因鼻过敏而感到困扰吗？ ①否 ②是

6.2请问是否连续超过1个月的时间都感到困扰呢？ ①否 ②是

7． 在过去的1年中，您曾经出现气喘或胸部发出鸣笛声吗? ①否 **请转问题8.** ② 是

7.1当发出气喘音时，您曾感到喘不过气来吗？ ①否 ②是

7.2 您不患感冒时，曾有过气喘或胸部发出鸣笛声吗? ①否 ②是

8． 在过去的1年中，您曾经出现过以下情况吗？(**可多选**)

①在醒来时感到胸部气闷 ②因气短而醒来 ③因咳嗽而醒来 ④都没有 ⑤不清楚

9． 您是否经常从胸部咳痰出来呢？①否 ②是 每年超过3个月吗？①否 ②是

10．您是否有过哮喘？ ①否 **请转问题11.**  ②是

10.1 您第一次哮喘发作时的年龄是(请尽可能估计一下) 岁。

10.2 您曾因哮喘住过院吗？ ①否 ②是

10.3 过去的12个月内您发作过哮喘吗？ ①否 ②是

10.4 您目前正在服用治疗哮喘的药物（包括吸入剂、喷雾剂或药片）吗? ①否 ②是

11．您曾经有过持续了6个月以上的发痒皮疹吗？ ①否 **请转问题12.**  ②是

11.1 在过去的12个月里您得过发痒的皮疹吗？ ①否 ②是

11.2 皮疹只影响您的双手吗？ ①否 ②是

12．您有过其它类型的皮肤过敏吗？ ①否 ②是

13．您曾经在服用止痛药后3个小时内感到呼吸困难吗？ ①否 ②是；止痛药的名称是

14．您经常会胃部反酸或胃部有烧灼感吗 ？ ①从不 ②偶尔 ③经常 ④每天

15. 您是否患过以下疾病？（**可勾选多个选项**，如有其它重要病史请给出具体疾病名称。）

①糖尿病 ②高血压 ③冠心病 ④高脂血症 ⑤痛风 ⑥慢性阻塞性肺病 ⑦ 癫痫

⑧恶性肿瘤（癌或肉瘤） ⑨其它 ⑩以上疾病都没有

**第三部分 生活方式等有关影响因素调查**

1. 现在或过去您是否曾有1年以上时间平均每天至少吸1支香烟？ ①否 **请转问题2.** ② 是

1.1您大约 岁开始吸烟。如果已停止吸烟，_____岁停止

1.2 现在或戒烟前您平均一天吸多少支烟？

　①1～5支/天 ②6～10支/天 ③11～20支/天 ④20支/天以上

2. 与您一起居住或工作的人中，有人吸烟吗?

①无 **请转问题3.**  ②有1人 ③有2人 ④有3人以上

2.1您每周被动吸烟（二手烟）的时间是：①每周1～9小时 ②每周10～39小时 ③每周40小时以上

2.2您累积被动吸烟 年；如果已脱离被动吸烟环境，已脱离了 年。

3. 您是否喝酒？①从不喝 **请转问题4.** ②很少喝 　 ③经常喝

3.1 如果喝酒，主要喝的种类是什么？①啤酒 ②红酒 ③白酒 ④其他

3.2 如果经常喝，从开始喝酒到现在有 年，

3.3 现每周酒量为 ①半斤以下 ②半斤-1斤 ③1斤-１斤半 ④１斤半以上

4. 您经常吃早餐吗？①从不吃 ②偶尔吃 ③经常 ④每天

5. 您经常体育锻炼吗？①从不　 ② 1-4次/月 ③ 5-8次/月 ④ 9-12次/月 ⑤ 12次以上/月

6. 您认为环境污染对您的健康影响有多大？①无影响 ②较小 ③一般 ④较大 ⑤极大

7. 您的家里有养：狗，猫，鸟或其他宠物吗？ ①否 ②是

8. 您的家里铺大片的地毯或整个房间都铺地毯吗？ ①否 ②是

9. 您工作的地方铺大片的地毯或整个房间都铺地毯吗？ ①否 ②是

10.您从事的工作有粉尘接触吗? ①否 **请转问题11.** ②是

10.1 如果您从事的工作有粉尘接触，请说明接触的粉尘类别（可多选）：

| ①矽尘 | ②水泥尘 | ③石棉尘 | ④化纤尘 |
| --- | --- | --- | --- |
| ⑤人造矿物纤维或玻璃纤维尘 | ⑥煤尘 | ⑦其他无机尘 | ⑧甘蔗尘 |
| ⑨棉尘 | ⑩麻尘 | ⑾粮食尘（谷物尘） | ⑿木尘 |
| ⒀动物皮毛尘 | ⒁其他有机尘 | ⒂不知道 |  |

10.2 请具体说明您接触上述粉尘的程度： ①轻微 ②中等 ③严重

10.3 您接触上述粉尘的年数：总共 年(少于 1 年填“0”)

10.4 您脱离这些粉尘的年数： 年(少于 1 年或者目前还在接触填“0”)

11. 您从事的工作有无接触有害气体或有害化学物质? ① 否 **请转问题12.**  ② 是

11.1 如果有，请选出您接触的有害物质（可多选）

①铅 ②苯系物 ③锰 ④二氧化硫 ⑤氮氧化物 ⑥一氧化碳 ⑦化学溶剂

⑧甲醛 ⑨不知道 ⑩其他

11.2 请具体说明您接触上述化学物质的程度：①轻微 ②中等 ③严重

11.3 您总共接触上述有害化学物质的年数： 年(少于 1 年填“0”)

11.4您脱离这些有害化学物质的年数： 年(少于 1 年或者目前还在接触填“0”)

12. 您会经常呆在潮湿或发霉的室内空间吗？ ①从不 ②偶尔 ③经常 ④每天

13. 在过去的一个月，您有多少次吃辛辣食物? ①从未或几乎未吃（每周少于一次）  **请转问题14.**

②每周 1-2 次 ③每周 3-4 次 ④每周 5-6 次 ⑤每天或几乎每天

13.1 您大约从什么时候开始习惯性地吃辛辣食品(最少每周一次)? 岁

13.2 您喜欢吃的辛辣食品的程度?  ①微辣 ②较辣 ③很辣

14. 您的住处主要使用下列哪一种燃料?

①天然气 ②液化石油气 ③煤／煤球／煤饼 ④柴/木炭 ⑤电 ⑥其他

15.您家厨房的炉子有安装烟囱或排烟设备吗? ①否 ②是

16.夏天您家或者工作的地方经常开冷气吗？

①没有 ②偶尔（每周少于两天）③每周 3－4 天 ④每周5－6天 ⑤每天或几乎每天

17.冬天您家是否取暖? ①否 **请转问题18.** ②是

17.1.您家取暖**主要**使用下列哪一种燃料（**只选一种燃料**）？

①中央供暖 ②气体燃料 ③煤／煤球／煤饼 ④柴/木炭 ⑤电 ⑥其他

18.您每天睡眠大约 小时。您的睡眠质量属于以下哪一种情况呢？

①睡眠质量较好 ②中间经常醒 ③入睡困难 ④早醒 ⑤梦游 ⑥其它 。

19.请根据您最近1年的饮食习惯，填写您吃下列各种食物的次数。（请在对应的框内上打“√”）

| 食物品种 | 吃的次数 | | | | | |
| --- | --- | --- | --- | --- | --- | --- |
|  | ①  每月少于１次 | ②  每月1～3次 | ③  每周1～2次 | ④  每周3～4次 | ⑤  每周5～6次 | ⑥  每周6次以上 |
| 鱼或海鲜 |  |  |  |  |  |  |
| 红肉（指猪肉、牛肉等） |  |  |  |  |  |  |
| 蔬菜 |  |  |  |  |  |  |
| 水果 |  |  |  |  |  |  |
| 豆制品 |  |  |  |  |  |  |
| 蛋类 |  |  |  |  |  |  |
| 奶和奶制品 |  |  |  |  |  |  |
| 咖啡 |  |  |  |  |  |  |
| 茶 |  |  |  |  |  |  |
| 饮料 |  |  |  |  |  |  |
| 冰淇淋或其他冷冻甜品 |  |  |  |  |  |  |
| 方便面、美式快餐(如麦当劳、肯德基） |  |  |  |  |  |  |
| 口服维生素 |  |  |  |  |  |  |

***谢谢您的合作！***

*本调查表是由比利时根特大学上呼吸道实验室提供的有关慢性鼻窦炎的流行病学调查表修改而来，特致谢。*
